# Clinical Applications of the Photopic Negative Response to Optic Nerve and Retinal Diseases

**DOI:** 10.1155/2012/397178

**Published:** 2012-10-24

**Authors:** Shigeki Machida

**Affiliations:** Department of Ophthalmology, Iwate Medical University School of Medicine, 19-1 Uchimaru, Iwate, Morioka 020-8505, Japan

## Abstract

The photopic negative response (PhNR) in response to a brief flash is a negative-going wave following the b-wave of the cone electroretinogram (ERG) that is driven by retinal ganglion cells (RGCs). The function of RGCs is objectively evaluated by analysing the PhNR. We reviewed articles regarding clinical use of the PhNR. The PhNR was well correlated with the visual sensitivity obtained by standard automated perimetry and morphometric parameters of the inner retina and optic nerve head in optic nerve and retinal diseases. Moreover, combining the PhNR with focal or multifocal ERG techniques enables the objective assessment of local function of RGCs. The PhNR is therefore likely to become established as an objective functional test for optic nerve and retinal diseases involving RGC injury.

## 1. Introduction

Retinal ganglion cells (RGCs) are selectively or preferentially damaged by diseases of the optic nerve and inner retina. Currently, there are surprisingly few methods to quantify RGC function. Visual field testing is used to determine visual function in patients with glaucoma and optic nerve disease, but it produces abnormal findings in the event of damage anywhere along the anterior visual pathway. Accordingly, this test method is not necessarily capable of selectively determining RGC function.

Objective tests of RGC function include visual evoked potentials (VEPs) and pattern electroretinograms (PERGs). The VEP measures potentials generated by the visual cortex, so, like visual field testing, it cannot directly measure RGC function. The PERG, on the other hand, reflects RGC function but still yields abnormal findings in patients with damage to the middle and outer layers of the retina. Standard ERGs must be recorded simultaneously in order to evaluate the function of the middle and outer retinal layers. Moreover, special equipment and refractive correction are required to perform this electrophysiological test.

**Figure 1:**
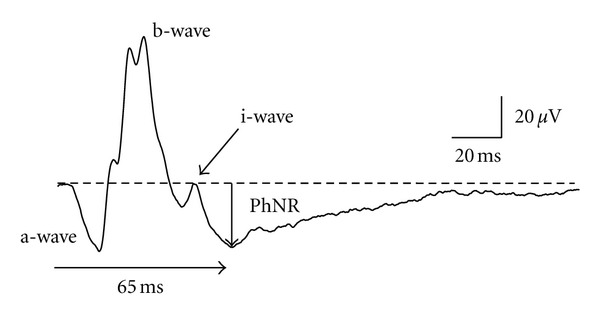
A representative waveform of the cone electroretinogram recoded from a normal subject by red stimuli on a blue background. PhNR: photopic negative response.

The standard ERG is conventionally thought to reflect electrical potentials mainly from photoreceptors and bipolar cells (or Müller cells). Recently, however, it was discovered that the RGC potentials contribute to the cone-driven ERG [1] in the form of the photopic negative response (PhNR) [2]. The PhNR in response to brief stimuli is the negative-going wave following the b-wave of the cone response (Figure 1). An advantage of the PhNR is that it can be recorded using a conventional ERG recording device. Furthermore, the PhNR is a component of the cone ERG, so a- and b-waves can be recorded simultaneously enabling the function of middle and outer retinal layers to be evaluated at the same time. This benefit is not available when assessing RGC function with the conventional means of the VEP or PERG. In addition, refractive corrections are not required when recording the PhNR. This simple recording and evaluation of the PhNR opens the way for clinical applications. The present paper therefore describes the potential clinical use of the PhNR in diseases of the optic nerve and inner retina.

## 2. Basic Research on the PhNR

### 2.1. Discovery of PhNR in Monkeys

RGC component in the cone ERG was discovered by Viswanathan et al. in 1999 [2]. They reported that the PhNR following the b-wave of the cone ERG disappeared from eyes of macaques after intravitreal injection of tetrodotoxin (TTX) which blocks voltage-gated sodium channels and thus blocks action potentials produced by RGCs and spiking amacrine cells [3, 4]. They also demonstrated that PhNR amplitudes were decreased in glaucomatous eyes with laser-induced ocular hypertension in monkeys. These experimental results implied that the PhNR arises from RGCs and/or their axons. However, one may have question why spiking action potentials produced by RGCs shape a slow negative waveform. Experimental evidence suggests that glial mediation generates the PhNR: an intravitreal injection of Ba^2+^ blocks K^+^ current in glia cells with the subsequent elimination of the PhNR in cats [5]. This suggests that glial mediation could contribute to shaping waveform of the PhNR.

Caution is needed when attempting to determine the origin of the PhNR because of its species specificity. In cat [6], monkeys [2], and humans [7] it derives from RGCs, but in animals such as rodents it originates from amacrine cells [8, 9]. The scotopic threshold response (STR) [10] which is elicited by very dim light under dark adaptation is a functional indicator of RGCs in rodents [8]. In rodents, the STR consists of positive and negative components. The positive STR is more affected by RGC damage than the negative STR [8].

### 2.2. PhNR Recording Conditions

The International Society for Clinical Electrophysiology of Vision (ISCEV) recommends that cone ERGs be recorded using white-flash stimuli on a white background light (“white-on-white”; W/W) [11]. On the other hand, Viswanathan et al. [2], who published the first study on the PhNR, used red-flash stimuli on a blue background (“red-on-blue”; R/B) to record the PhNR. The colored flash stimuli and background are generated by light-emitting diodes (LEDs), giving them a narrow, half-width spectrum. It has been shown that R/B elicited the PhNR with more RGC responses than did W/W especially in the low and intermediate stimulus range [12]. While future studies are needed to determine the ideal stimulus conditions for evaluating PhNR, RGC-derived potentials are reflected in the PhNR recorded under either W/W or R/B conditions.

The S-cone ERG can be recorded by blue stimuli under a yellow background to suppress responses of the M- and L-cones. The PhNR of the S-cone ERG is reported to be especially vulnerable to glaucoma and diabetic retinopathy [13, 14].

### 2.3. PhNR in Focal ERG (Focal PhNR)

**Figure 2:**
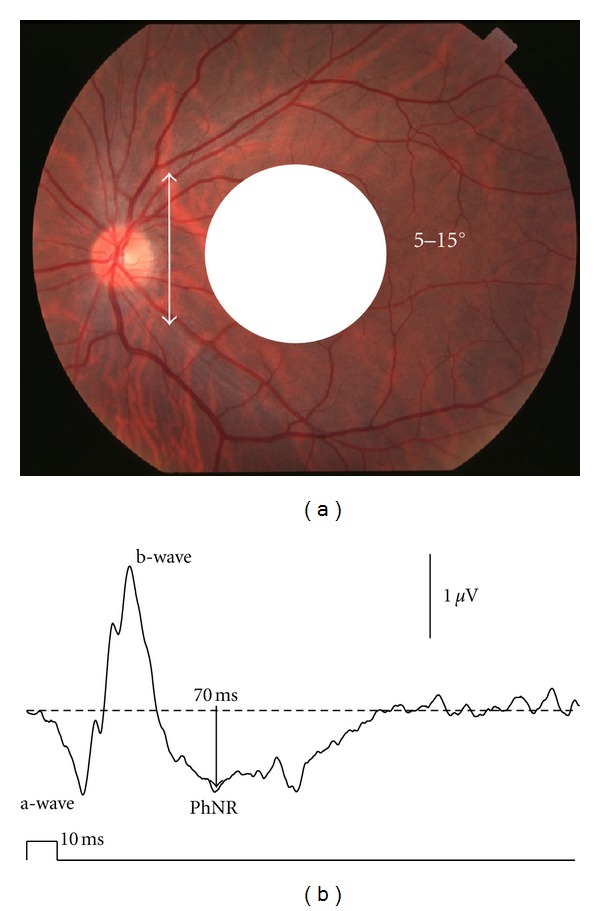
(a) Stimulus spot centered on the macula for recording the focal macular electroretinogram (ERG). (b) The focal macular ERG consists of the a- and b-waves and photopic negative response (PhNR).

The focal ERG developed by Miyake et al. [15] consists of the a-wave, b-wave, oscillatory potentials, and PhNR (focal PhNR) (Figure 2). The focal ERG stimulus system is built into an infrared fundus camera and delivers stimuli onto the local retina using a 5–15° stimulus spot while viewing the ocular fundus (Figure 2(a)). This allows the device to acquire very reliable data from the macula, even in individuals with poor fixation. Colotto et al. [16] firstly applied the focal PhNR to patients with glaucoma, although they used a different recording system from Miyake's one.

Like the PhNR obtained by full-field stimuli (full-field PhNR), the focal PhNR also disappeared following intravitreal injection of TTX in monkey eyes [17]. Moreover, the amplitude of both focal and full-field PhNR was reduced in patients with optic nerve atrophy [18]. Based on these results, the focal PhNR is also believed to originate from RGCs of the local retinal area.

### 2.4. PhNR in Multifocal ERG (Multifocal PhNR)

In the standard multifocal ERG, the stimulus frequency is set high at 75 Hz. A stimulus is delivered once every 13.3 msec, making it hard to record the entire part of the PhNR, which has a peak latency of approximately 70 msec. The amplifier settings also eliminate the most part of PhNRs because the low-cut filter is usually set at 10 Hz. It is therefore essential to reduce the stimulus frequency and low-cut filter in order to record PhNRs with the multifocal ERG.

**Figure 3:**
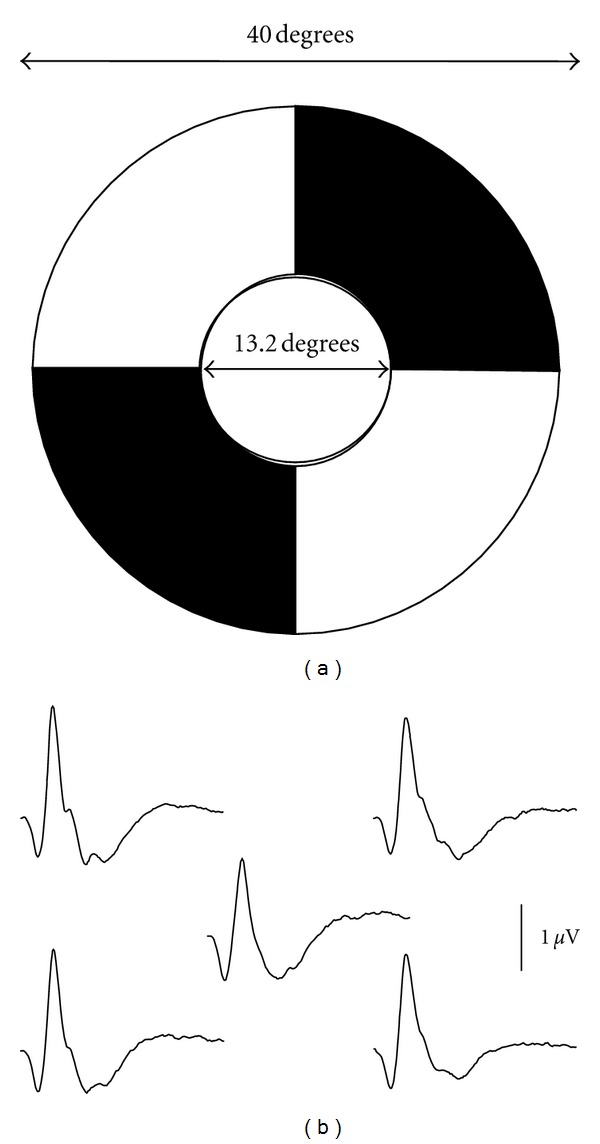
(a) A dartboard pattern of stimuli for recording the multifocal electroretinogram (ERG). (b) Normal waveforms of multifocal ERG recorded from each element.

With this in mind, we attempted to record the multifocal ERG by setting the stimulus frequency at 6.25 Hz and low-cut filter at 3 Hz using a stimulus display with a dartboard pattern (Figure 3(a)). The respective patterns are inverted from white to black and vice versa in a pseudorandom sequence. Waveforms resembling focal ERG containing PhNRs were obtained from each element (Figure 3(b)). Kaneko et al. [19] have demonstrated that the multifocal PhNR amplitudes were deteriorated by optic nerve atrophy, indicating that the multifocal PhNR also originates from RGCs. While the clinical significance of the PhNR in the multifocal ERG is a topic for future research, the use of the multifocal ERG may allow us to evaluate the RGC function in each part of the retina in the posterior pole of the ocular fundus.

### 2.5. Evaluation of PhNR

The PhNR is a relatively slow wave modified by positive i-waves, so its peak is often difficult to determine. This in turn makes it difficult to accurately evaluate peak latency of the PhNR. Measuring PhNR amplitude in healthy individuals at 5 ms intervals yielded a maximum amplitude at 65 ms for full-field PhNR and 70 ms for focal PhNR. Full-field and focal PhNR amplitudes at 65 and 70 ms, respectively, have therefore been measured from the baseline (Figures 1 and 2(b)). However, the waveform of the PhNR changes with recording conditions, such as stimulus parameters and amplifier settings. Adequate settings of low-cut filters are required for reliable recordings of the PhNR by avoiding low-frequency drift of the baseline. Therefore, each laboratory has to choose a fixed implicit time for measuring the PhNR amplitude based on own data. This method of measuring PhNR amplitude is believed to be the simplest and least biased, but there is still no consensus on a uniform method. In fact, various studies use different measurement procedures, so care needs to be exercised in this regard.

## 3. Clinical Applications of PhNR

### 3.1. Optic Nerve Atrophy

The PhNR has been studied in patients with optic nerve atrophy induced by trauma [20], gene mutation [21], inflammation [22], compression [23, 24], and ischemia [25]. In these studies, the PhNR has been shown to be selectively or predominantly affected by these diseases. In our previous study examining changes in the cone ERG of patients with traumatic optic neuropathy, we found that ERG was virtually normal immediately after the injury but that the PhNR amplitude alone decreased selectively upon the onset of optic disc atrophy and optic nerve pallor [20]. This finding suggests that PhNRs reflect the state of RGCs and do not appear abnormal when the lesion is confined to the optic nerve behind the eye and when intraocular RGCs are normal.

**Figure 4:**
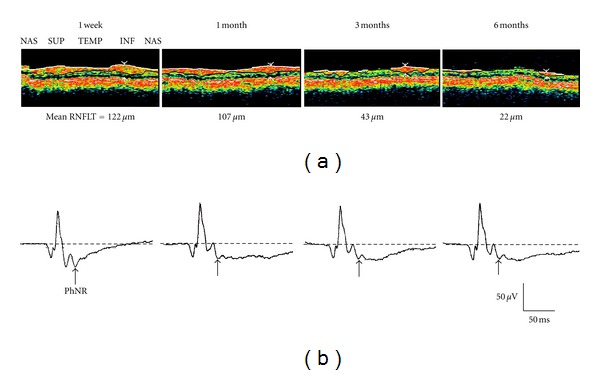
(a) Retinal nerve fiber layer thickness (RNFLT) around the optic nerve head measured by optical coherence tomography in a patient with traumatic optic neuropathy at 1 week and 1, 3, and 6 months after the injury. (b) Cone electroretinograms recorded simultaneously. PhNR: photopic negative response (reproduced with permission from [20]).

We previously conducted a prospective study of the relationship between the PhNR amplitude following traumatic optic neuropathy and retinal nerve fiber layer thickness (RNFLT) surrounding the optic disc [20] (Figure 4). Even when RNFLT was maintained at 1 month after the injury, the PhNR amplitude declined dramatically. This decrease in the PhNR amplitude preceded thinning of RNFLT. In other words, RGCs undergo a functional decline before the occurrence of morphological changes.

The full-field PhNR is believed to be characteristic of overall RGC function and could therefore be used to evaluate function in optic nerve diseases with extensive RGC damage. However, many patients with optic nerve disease have central scotoma in which extensive RGC injury is not necessarily present. Therefore, if the focal ERG could be used to determine the RGC function in the local retina, this could conceivably lead to improvements in diagnostic capability.

**Figure 5:**
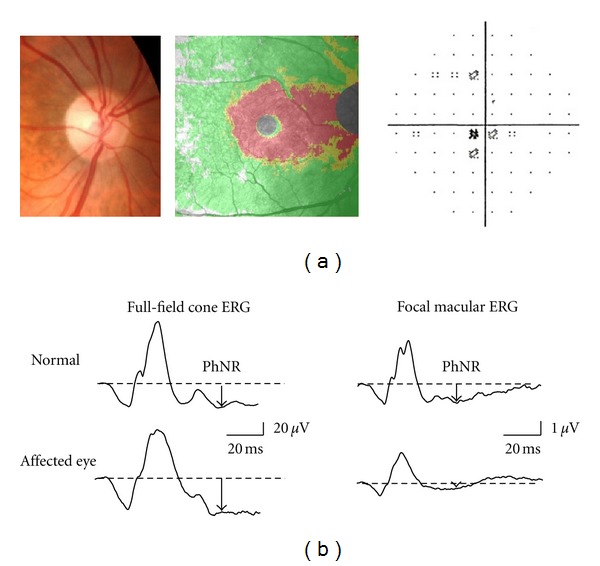
A representative case of optic nerve atrophy. (a) Optical coherence tomography detected abnormally thinning area of ganglion cell complex thickness (indicated by red area). Standard automated perimetry demonstrated central scotoma. (b) The full-field cone and focal macular electroretinograms (ERGs) recorded from a normal subject and the representative case. PhNR: photopic negative response.

In our previous study in which the full-field cone and focal macular ERGs were recorded in patients with localized optic nerve atrophy, we compared the full-field and focal PhNRs [18]. In a representative case, a slight pallor was observed on the temporal side of the optic disc corresponding to abnormally thinning area of ganglion cell complex (GCC) thickness in the central area of the ocular fundus (indicated by red areas), and central scotoma was also observed (Figure 5(a)). GCC consists of the retinal nerve fiber, ganglion cell, and inner plexiform layers. The full-field PhNR amplitude remained normal, but the focal PhNR amplitude diminished considerably (Figure 5(b)). This finding implies that the focal PhNR is an indicator of local RGC damage. Previously, we examined both central and diffuse types of optic nerve atrophy [18]. The central type manifests as central scotoma whereas the diffuse type is characterized by a diffuse decrease in the visual sensitivity. In patients with diffuse-type of optic nerve atrophy, both focal and full-field PhNR amplitude fell below the lower limit of normal. Meanwhile, those with the central type of optic nerve atrophy exhibited normal full-field PhNR amplitude but a decline in the focal PhNR amplitude to below the normal limit. These results imply that the focal PhNR is useful in diagnosing localized optic nerve atrophy.

### 3.2. Glaucoma

**Figure 6:**
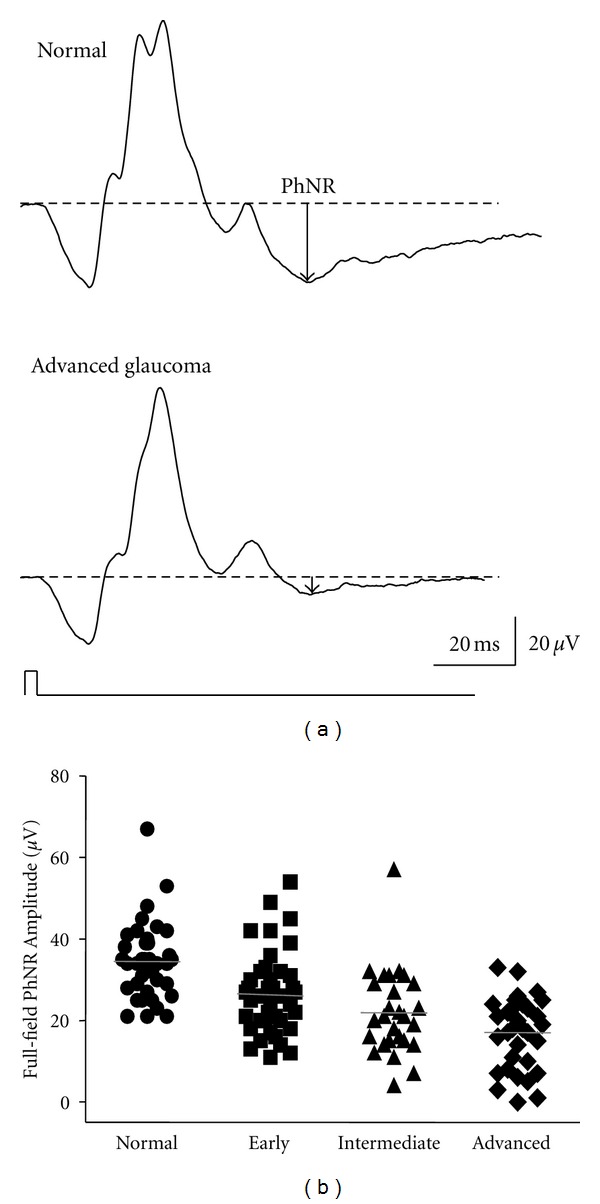
(a) Representative waveforms of the full-field cone electroretinogram recorded from a normal subject and a patient with advanced glaucoma. (b) PhNR amplitudes were plotted for normal subjects (*n* = 42) and patients with early (*n* = 41), intermediate (*n* = 28), and advanced glaucoma (*n* = 34). PhNR: photopic negative response (reproduced with permission from [27]).

Glaucoma is a typical disease involving damage to RGCs. As shown in Figure 6(a) in a glaucomatous eye, the cone a- and b-wave amplitudes are normal but the PhNR amplitude of the full-field cone ERG is diminished [7, 26, 27]. This decrease in full-field PhNR amplitude grew as the glaucoma became more severe (Figure 6(b)). It was also reduced as the mean deviation determined by static automated perimetry (SAP) worsened [26, 27]. Significant correlations have been identified between full-field PhNR amplitude and the morphological indicators of RNFLT surrounding the optic disc, the optic disc rim area, and cup/disc area ratio [26, 27]. Put simply, the full-field PhNR is a feasible indicator of glaucoma-induced functional and morphological impairment of RGCs.

The sensitivity and specificity to detect glaucoma by full-field PhNRs were 77% and 90%, respectively. However, the sensitivity declined to 57 [26] or 38% [27] for early-stage glaucoma, so the full-field PhNR was not a viable method of detecting the disease at the early stage. In early-stage glaucoma the RGC axons are locally damaged, so the full-field PhNR (which reflects the RGC function of the entire retina) is not suitable for determining localized RGC injury. On the other hand, changes in early-stage glaucoma could be detected if it were possible to record focal PhNRs from damaged RGCs using the focal ERG.

**Figure 7:**
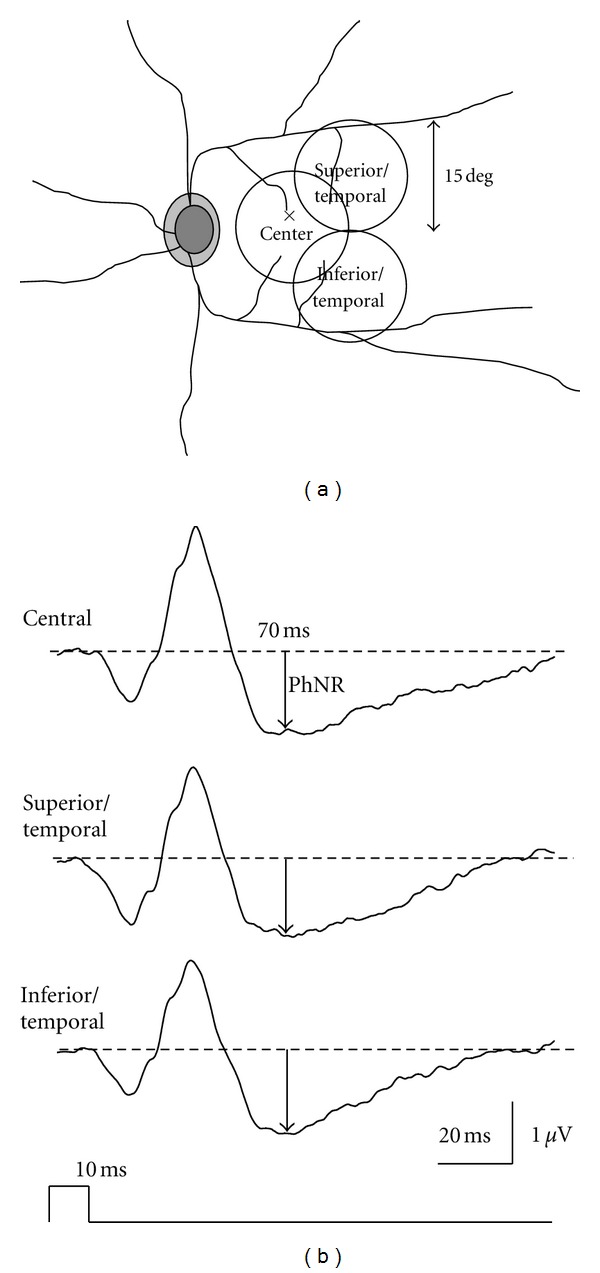
(a) Recording sites of focal electroretinograms. (b) Representative waveforms of the focal electroretinogram recorded from a normal subject. PhNR: photopic negative response.

**Figure 8:**
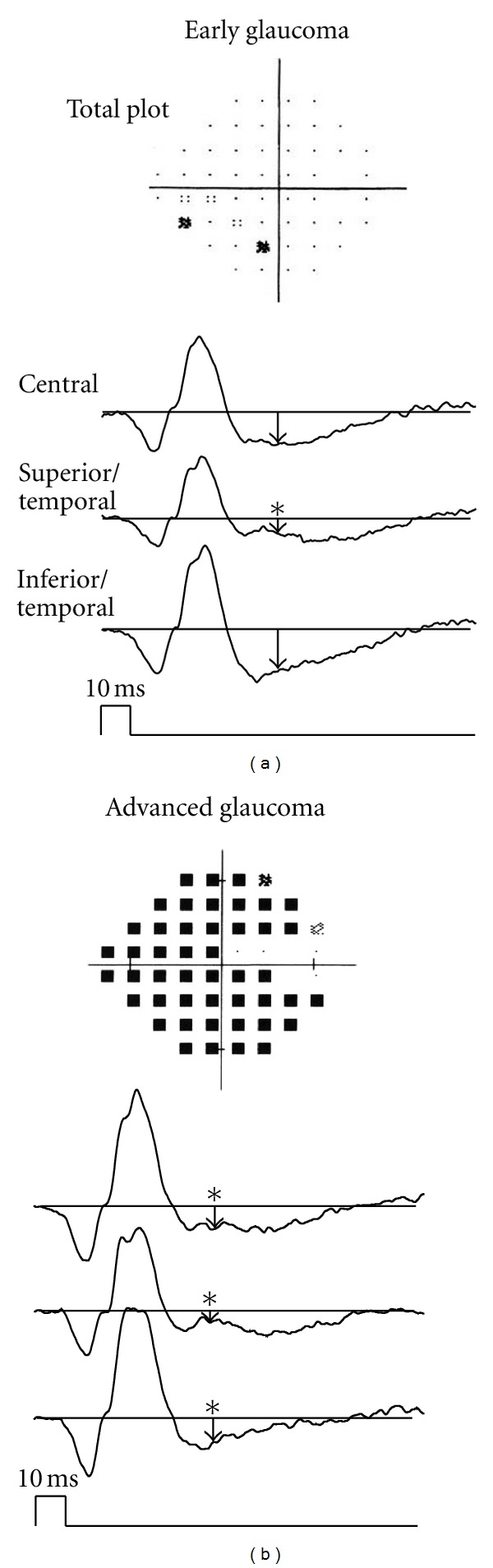
Representative cases of early (a) and advanced (b) glaucoma (reproduced with permission from [28]).

Glaucoma-induced RGC damage begins in the paramacular region (Bjerrum's area). Therefore, detection of early glaucomatous lesions would be difficult if the focal ERG was recorded only in macular region. With this in mind, we recorded the focal ERG not only in the macular region but also in the superotemporal and inferotemporal areas of the macula (Figure 7(a)) [27, 28, 31]. With this protocol, it is possible to record evaluable focal PhNRs from all stimulus sites (Figure 7(b)). As seen in the representative case of early glaucoma with a visual field defect in the inferonasal quadrant, the only decrease in the focal PhNR amplitude was seen in the superotemporal retina corresponding to the visual field defect (indicated by an asterisk, Figure 8(a)). Thus, the focal PhNR amplitude only decreased in 1 or 2 of the 3 recording sites in patients with early or intermediate glaucoma. When the focal PhNR amplitude was abnormally reduced in either recording sites, the eyes were defined to be glaucomatous. Consequently, sensitivity and specificity were no less than 90% even for early glaucoma when this diagnostic criterion was employed. In advanced glaucoma with severe visual field defects, the focal PhNR amplitude decreased at all recording sites (indicated by asterisks, Figure 8(b)).

The high sensitivity of the focal PhNR indicates that the focal PhNR is more suitable than the full-field PhNR for detecting functional loss of early glaucoma. However, the signal of the ERG is much smaller than that of the full-field ERG and thus the signal/noise ratio is smaller for the focal ERG, raising a possibility that the focal PhNR is less reliable than the full-field PhNR. Intersession variability is represented by the coefficients of variation (CV = standard deviation/mean *×* 100), and it was higher for the focal PhNR than for the full-field PhNR [26, 31]. In addition, variations of the PhNR amplitude among individuals were greater for the focal PhNR amplitude than for the full-field PhNR amplitude [26, 31]. However, this disadvantage of the focal PhNR can be reduced by using the amplitude ratio of the PhNR to the b-wave amplitude [31]. Therefore, the PhNR/b-wave amplitude ratio is recommended for measuring the effectiveness of the focal PhNR.

**Figure 9:**
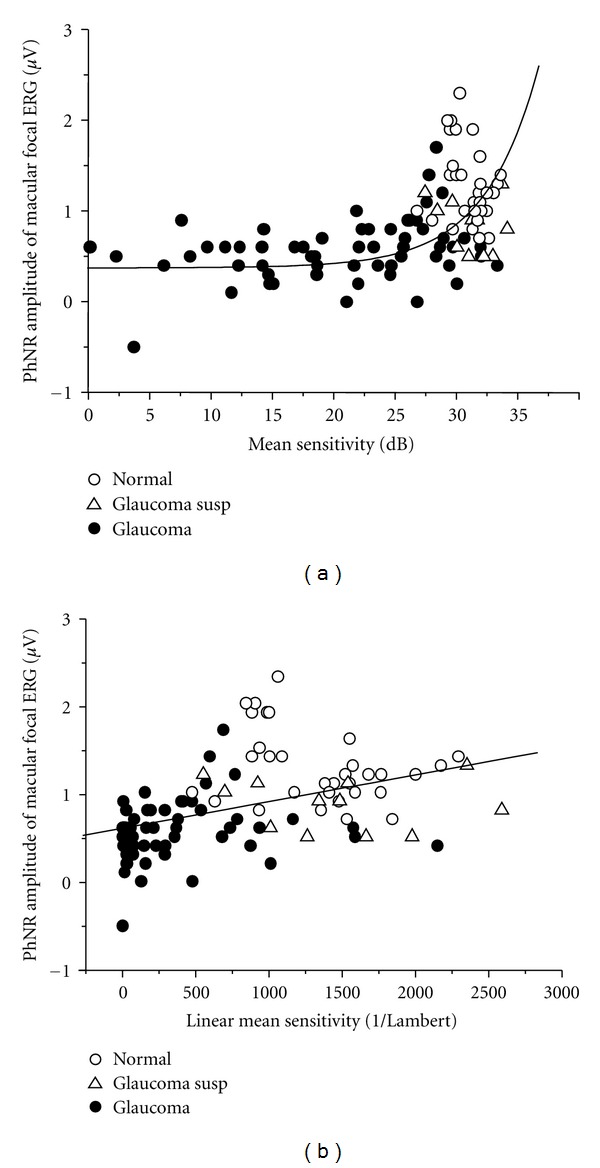
(a) The PhNR amplitude of the focal macular electroretinogram was plotted against mean visual sensitivity (dB) obtained by standard automated perimetry 10-2 program. The fitting curve was obtained by the equation based on Hood model [29, 30].  (b) The mean visual sensitivity (dB) was converted to a linear value (1/Lambert). PhNR: photopic negative response (reproduced with permission from [31]).

The relationship between the focal PhNR amplitude and visual sensitivity (dB) determined by SAP at either ERG recording site was nonlinear [31]. That is, even a slight drop in the visual sensitivity (dB) resulted in a major decline in the focal PhNR amplitude. Furthermore, even when the visual sensitivity (dB) fell, the focal PhNR amplitude remained nearly unchanged (Figure 9(a)). These findings suggest that the focal PhNR is a useful indicator in the early diagnosis of glaucoma. Put differently, the focal PhNR is unsuitable for use in following up patients with intermediate or advanced glaucoma, and the visual sensitivity (dB) should instead be used for this purpose. The curve in Figure 9(a) was fit to the following equation based on the Hood model [29, 30]:

(1) $$\begin{array}{c} R={A\times 10}^{0.1(S-30)}+B, \end{array}$$

where *R* is the focal PhNR amplitude; *A* is the focal PhNR amplitude of normal RGCs; *S*is mean of visual sensitivity determined by SAP; *B* is the basal level of the focal PhNR amplitude when a patient has lost sensitivity to light.

The fact that the focal PhNR amplitude and visual sensitivity (dB) have a nonlinear relationship can be attributed to the fact that dB is a logarithmic value that can be expressed as follows:

(2) $$\begin{array}{c} \text{dB}=10\log\left(\frac{1}{\text{Lambert}}\right)∴\frac{1}{\text{Lambert}}=10^{0.1\times \text{dB}}. \end{array}$$

When converting the visual sensitivity (dB) from a log value to a linear value (1/Lambert) using the previously mentioned equation, the relationship between the focal PhNR amplitude and visual sensitivity became linear (Figure 9(b)). The focal PhNR amplitude is also significantly correlated with local changes in RNFLT, rim area, cup/disc area ratio, or GCC thickness [16, 32, 33]. This indicates that the focal PhNR reflects the morphological changes associated with glaucoma of local area of the retina or optic disc.

### 3.3. Inner Retinal Diseases

**Figure 10:**
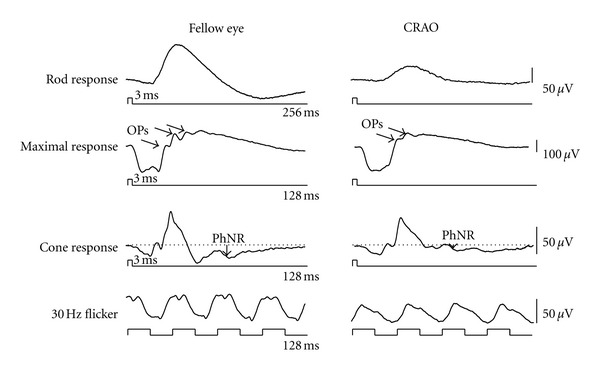
Representative waveforms of the full-field electroretinogram recorded from unaffected fellow and affected eyes with central retinal artery occlusion (CRAO). OPs: oscillatory potentials, PhNR: photopic negative response.

**Figure 11:**
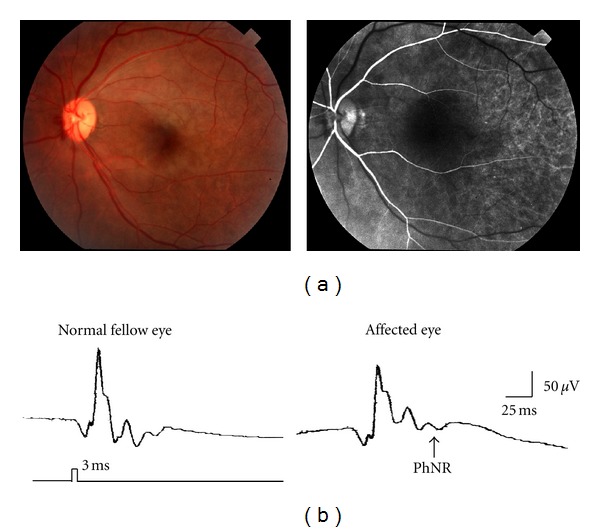
(a) Early recanalization of blood flow was seen by fluorescein angiogram. (b) The PhNR amplitude was considerably reduced in the affected eye. PhNR: photopic negative response.

Depression of the b-wave amplitude with leaving the a-wave unchanged is a well-known ERG finding in patients with central retinal artery occlusion (CRAO).  Figure 10 shows the full-field ERG recorded from the fellow eye and affected eye of a CRAO patient. Focusing on the cone response, we can see that the full-field PhNR amplitude was dramatically decreased by CRAO [34]. When the respective wave amplitudes of the CRAO eye are expressed as a ratio of those of the healthy fellow eye (i.e., amplitude ratio), it became apparent that the full-field PhNR amplitude was predominantly lower than the a- and b-wave amplitudes. This is consistent with the pathological finding that damage to the inner retinal layers is greatest among CRAO patients. When early recanalization of blood flow occurs in CRAO patients, fundus findings may be subtle (Figure 11(a)), necessitating differential diagnosis from acute optic nerve diseases. Even in these patients, however, the full-field PhNR amplitude was considerably depressed (Figure 11(b)). It has been reported that the full-field PhNR could be used to evaluate degree of ischemia or visual prognosis in patients with CRAO [35, 36].

Diminished PhNR amplitude of the full-field ERG is also observed in central retinal vein occlusion [37] and early diabetic retinopathy [38, 39]. In other words, the full-field PhNR may also be useful in the diagnosis and functional assessment of ischemic retinal diseases.

**Figure 12:**
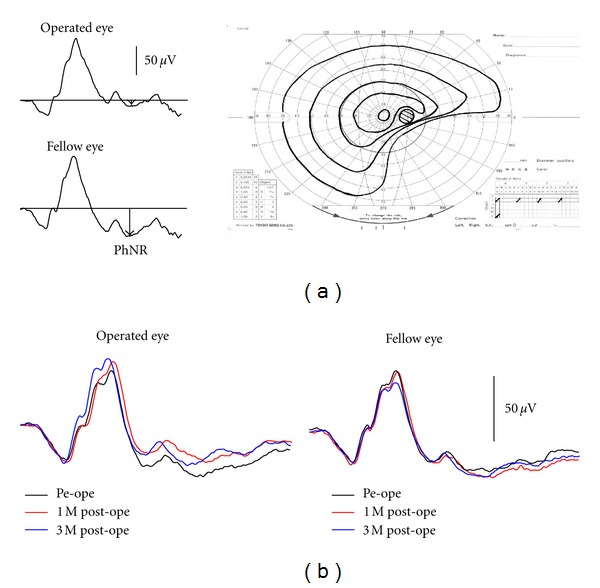
(a) Representative case of macular hole who complained of visual field defect in the operated eye after surgery. (b) Changes of the full-field cone electroretinograms recorded from a patient with a macular hole pre- and postoperatively. PhNR: photopic negative response.

Indocyanine green (ICG) is used during macular hole surgery to visualize inner limiting membrane. The toxicity of ICG on RGCs has previously been demonstrated in an animal study [40]. The PhNR amplitude was reduced in patient who has developed visual field defects following macular hole surgery (Figure 12(a)). Ueno et al. [41] reported that the full-field PhNR was significantly reduced even in patients without developing visual field defects after surgeries.  Figure 12(b) shows the time-course changes in the cone ERG before and after macular hole surgery. In the fellow eye there was virtually no change in the ERG, but in the operated eye there was a delay in the b-wave peak and slight decline in the PhNR amplitude at 1 month after surgery. At 3 months after surgery, this delay in the b-wave peak disappeared but the PhNR amplitude remained mildly depressed. While the decline in PhNR amplitude is slight, it may indicate subclinical RGC damage incurred during vitreous surgery to repair macular holes.

## 4. Conclusions

The use of the PhNR has enabled objective evaluation of RGC function. The PhNR can also be measured in clinical settings due to the ease with which it can be recorded and evaluated. Moreover, combining the PhNR with focal or multifocal ERG techniques enables the objective assessment of local function of RGC. The PhNR is therefore likely to become established as an objective functional test for optic nerve and retinal diseases involving RGC injury. However, further studies on the prognostic value of the PhNR in these diseases are required to establish the clinical utility of this technique.
